# Hair Tourniquet Syndrome of Labia Minora

**Published:** 2015-05-01

**Authors:** Antonios Panagidis, Xenophon Sinopidis, Vasileios Alexopoulos, George Georgiou

**Affiliations:** 1 Department of Pediatric Surgery, Karamandanion Children’s Hospital, Patras, Greece; 2 Department of Pediatric Surgery, University Hospital, Patras, Greece

**Dear Sir,**

We present two uncommon cases of adolescent girls with hair-thread strangulation of the labia minora. The first 14-year-old girl presented with a painful pedunculated labial lump (Fig. 1). The lesion was covered with exudate. She was examined under sedation and found a coil of long hair forming a tourniquet around a labial segment. Thread removal resulted to immediate relief from pain, and gradual return to normal appearance. Another 10-year-old girl presented with a similar labial swelling. The recent experience of the first case led us straight to the problem. A long hair-thread was found at the neck of the lesion. Hair removal resulted in settling of the pain. The labial swelling subsided in few days.

**Figure:**
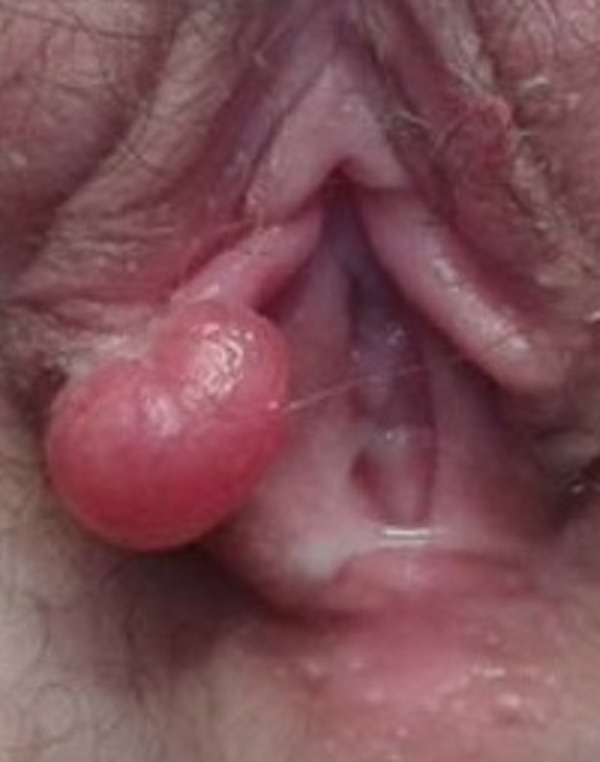
Figure 1: External genitalia inspection revealing a painful labial lump

Hair tourniquet syndrome (HTS), a strangulation of body appendages by a thread of hair, is a rare and often misdiagnosed condition which may result in accidental organ amputation [1]. Hair may wrap and create a tourniquet resulting in lymphatic obliteration, venous congestion, and arterial obstruction which occasionally lead to necrosis and amputation of the part involved. Hair tends to lacerate the skin or the mucosa, and embeds in deeper tissues [2]. Swelling of the strangulated appendage and the physical characteristics of hair contribute to progressive cutting of the soft tissues resulting in excruciate pain [3].

Female urogenital area is the most unusual location of HTS, involving primarily the clitoris [3]. In a review on 66 cases of HTS in children there were only two cases of clitoris strangulation, and one of labia majora [1]. In one case of clitoral HTS, hair from the pubic area and not from the head was involved [5]. Most cases of HTS are accidental, caused by the hair of the head; self-inflicted hair wrap should also be kept in mind [5]. Behavioral disorders have been discussed as predisposing factors [1-5]. Differential diagnosis of genital HTS includes any painful cystic lesion of the genital area [2, 4]. Hair-thread removal is not always easy, particularly in cases with long standing symptoms. Both our patients had an ordinary psychosocial profile.

## Footnotes

**Source of Support:** Nil

**Conflict of Interest:** None declared

